# Abrogating Metastatic Properties of Triple-Negative Breast Cancer Cells by EGFR and PI3K Dual Inhibitors

**DOI:** 10.3390/cancers15153973

**Published:** 2023-08-04

**Authors:** Ana Rita Garcia, Avilson Mendes, Carlos Custódia, Cláudia C. Faria, João T. Barata, Rui Malhó, Inês Figueira, Maria Alexandra Brito

**Affiliations:** 1 Research Institute for Medicines, Faculty of Pharmacy, Universidade de Lisboa, Av. Prof. Gama Pinto, 1649-003 Lisbon, Portugal; 2 Department of Pharmaceutical Sciences and Medicines, Faculty of Pharmacy, Universidade de Lisboa, Av. Prof. Gama Pinto, 1649-003 Lisbon, Portugal; 3 Instituto de Medicina Molecular João Lobo Antunes, Faculdade de Medicina, Universidade de Lisboa, Av. Prof. Egas Moniz, 1649-028 Lisbon, Portugal; 4 Department of Neurosurgery, Hospital de Santa Maria, Centro Hospitalar Universitário Lisboa Norte (CHULN), Av. Prof. Egas Moniz, 1649-035 Lisbon, Portugal; 5 BioISI—Biosystems and Integrative Sciences Institute, Faculty of Sciences, Universidade de Lisboa, Campo Grande, 1746-016 Lisbon, Portugal; 6 Farm-ID—Faculty of Pharmacy Association for Research and Development, Av. Prof. Gama Pinto, 1649-003 Lisbon, Portugal

**Keywords:** brain metastases, cell cycle, cell death, dual inhibitors, epidermal growth factor receptor, forkhead box P1, myocyte enhancer factor 2C, migration, phosphoinositide 3-kinase, triple-negative breast cancer

## Abstract

**Simple Summary:**

Triple-negative breast cancer (TNBC) is an aggressive BC that highly metastasizes to the brain, constituting a major hurdle and with poor survival after diagnosis. This strongly contributes to the lack of well-defined molecular targets, limiting effective TNBC therapeutic options. Nevertheless, TNBC has been associated with epidermal growth factor receptor (EGFR) overexpression and downstream phosphoinositide 3-kinase (PI3K) signaling activation. We hypothesized that blood–brain barrier (BBB)-permeant molecules with the predicted ability to inhibit the EGFR/PI3Kp110β pathway could be strong candidates to tackle metastatic TNBC. A screening of EGFR, PI3Kp110β, and dual inhibitors was performed to validate their efficacy in biological assays. We used TNBC cells with brain tropism to test the drugs’ effects in downstream players of the EGFR/PI3Kp110β pathway, as well as in cell death, morphology, proliferation, and migration. We found that two dual inhibitors presented the strongest anti-tumor effects, pointing to their usefulness in abrogating TNBC cells’ tumorigenic properties.

**Abstract:**

Triple-negative breast cancer (TNBC) is a devastating BC subtype. Its aggressiveness, allied to the lack of well-defined molecular targets, usually culminates in the appearance of metastases that account for poor prognosis, particularly when they develop in the brain. Nevertheless, TNBC has been associated with epidermal growth factor receptor (EGFR) overexpression, leading to downstream phosphoinositide 3-kinase (PI3K) signaling activation. We aimed to unravel novel drug candidates for TNBC treatment based on EGFR and/or PI3K inhibition. Using a highly metastatic TNBC cell line with brain tropism (MDA-MB-231 Br4) and a library of 27 drug candidates in silico predicted to inhibit EGFR, PI3K, or EGFR plus PI3K, and to cross the blood–brain barrier, we evaluated the effects on cell viability. The half maximal inhibitory concentration (IC_50_) of the most cytotoxic ones was established, and cell cycle and death, as well as migration and EGFR pathway intervenient, were further evaluated. Two dual inhibitors emerged as the most promising drugs, with the ability to modulate cell cycle, death, migration and proliferation, morphology, and PI3K/AKT cascade players such as myocyte enhancer factor 2C (MEF2C) and forkhead box P1 (FOXP1). This work revealed EGFR/PI3K dual inhibitors as strong candidates to tackle brain metastatic TNBC cells.

## 1. Introduction

Breast cancer (BC) is the most frequent malignancy in women, with an incidence of more than 2 million new cases and close to 700,000 deaths worldwide reported in 2020 [1], and a prediction of 3.2 million new cases per year in 2050 [2]. Triple-negative BC (TNBC) is one of the most aggressive subtypes, constituting about 15–20% of BC cases. Moreover, TNBC is characterized by the lack of hormone receptors (estrogen receptor and progesterone receptor) and human epidermal growth factor receptor 2 (HER2) expression [3,4], which has been correlated with a relatively poor patient outcome [5]. Among the different BC subtypes, it has been reported that TNBC primary tumors have a higher risk of metastasizing, being directly associated with the shortest overall survival [5]. Estimates indicate that 15% of BC patients develop brain metastases [6], a life-threatening condition with a 1-year survival rate of only 20% [7].

Within BC subtypes, TNBC presents the fewest therapeutic options due to the lack of well-defined molecular targets [8]. Nevertheless, the overexpression of tyrosine kinase receptors (TKRs), such as the epidermal growth factor receptor (EGFR), has emerged as associated with TNBC, enhancing tumor cells proliferation, migration, and invasiveness, comprising a potential therapeutic target [9,10]. The search for new drugs against TNBC has led to the development of anti-EGFR targeted therapeutic options such as antibodies and small molecule kinase inhibitors [11,12,13]. Unfortunately, some have demonstrated toxicity and low efficacy in monotherapy and in combination with chemotherapy, possibly explained by the coactivation and/or amplification of other TKRs that trigger compensatory signaling pathways or the acquisition of secondary EGFR point mutations [14,15].

A dysfunctional increase in EGFR catalytic activity can lead to a subsequent overactivation of downstream signaling cascades such as phosphatidylinositol 3-kinase (PI3K), which activation has been correlated with TNBC [16]. The PI3K signaling pathway has a vital role in the regulation of cell growth and survival in BC, and its molecular mechanisms have been explored [17,18,19]. Downstream PI3K is one of the most relevant players in the cascade, protein kinase B (also known as AKT). Once activated, AKT phosphorylates key substrates to regulate several signaling pathways that promote cell survival, growth, and proliferation. Therefore, in BC, PI3K/AKT pathway plays a fundamental oncogenic role, interacting with other canonical signaling pathways to drive tumor evolution and resistance to therapies [20,21] and modulating the expression of transcription factors, which play key roles in tumor cell migration and invasion [22,23]. Interestingly, the oncogenic activation of the p110β isoform (PI3Kp110β) was shown to be essential for tumor formation, including BC, and to be associated with recurrence rate. It deserves to be highlighted that PI3Kp110β-selective inhibitors are more specific in AKT deactivation than other PI3K isoform-specific inhibitors [24,25], rendering PI3Kp110β a potential target, namely to overcome cancer multidrug resistance [26].

PI3K/AKT signaling is important for the regulation of transcription factors such as forkhead box P1 (FOXP1) [27]. FOXP1 is a DNA-binding transcription factor that can act either as a tumor suppressor or as an oncogene [28,29]. Particularly in BC, FOXP1 overexpression was related to tumor proliferation [30]. The cell cycle and malignant transformation are regulated by D-type cyclins [31]. Among them is cyclin D2, which can be either overexpressed or under-expressed, depending on the type of cancer. As far as BC is concerned, cyclin D2 presents a low expression in BC cell lines, such as the widely used MDA-MB-231 [32]. Moreover, it was reported a lack of expression of cyclin D2 in primary breast carcinomas, in contrast to normal breast tissue [31,33] and other cancers [33]. Silencing of cyclin D2 expression was associated with hypermethylation of the promoter and suggested to be an early event in tumorigenesis and an indicator of poor prognosis [31,33].

The activation of EGFR induces p38 mitogen-activated protein kinase (MAPK) activation [34,35]. One downstream target of p38MAPK is myocyte enhancer factor (MEF)2C [34], a member of the MEF2 family of transcription factors [36]. MEF2C was found to be upregulated in BC, regulating different mechanisms of malignant cells such as migration, invasion, and proliferation [36]. MEF2C expression has been shown to increase during tumor progression and to increasingly translocate into the nucleus along brain metastatic growth in a mouse model [37]. Moreover, it was shown that MEF2C expression and nuclear translocation increase with disease severity in resected brain metastases from BC patients [38]. Thus, the search for EGFR and/or PI3K inhibitors able to abrogate tumorigenesis by modulating specific players in the signaling cascade, such as MEF2C and FOXP1, should be pursued.

Using computer-aided drug discovery tools such as quantitative structure-activity relation (QSAR) models and structure-based virtual screening, we previously searched for drug candidates with a predicted ability to inhibit the EGFR/PI3K pathway and to cross the blood–brain barrier (BBB) for glioma treatment [39]. These in silico studies led to the discovery of 18 candidate inhibitors for EGFR, 6 for PI3Kp110β, and 3 dual inhibitors of both targets, which efficacy against glioma cells was validated in parental and EGFR overexpressing cell lines [39]. Moreover, the most promising ones were further assessed for their BBB-permeation properties by quantification of each molecule in the “brain compartment” of a simplified in vitro model of the BBB, as well as for their tolerability by human BBB endothelial cells. Based on the promising results obtained [39] and on the fact that EGFR/PI3K signaling is common to both glioma and TNBC, we hypothesized that such small molecules might also be able to modulate key tumorigenic events in BC cells, particularly in those with brain tropism.

With this work, we aimed to disclose effective therapeutics for the highly metastatic TNBC, relying on EGFR and/or PI3K inhibitors and evaluating their potential to abrogate the downstream signaling cascade. Among the 27 small molecule inhibitors tested, two EGFR/PI3K dual inhibitors emerged as the most promising ones, with strong effects in modulating tumor cell migration, cell cycle, and proliferation, mechanisms of cell death, morphology, as well as in key players of PI3K/AKT cascade such as MEF2C and FOXP1. Overall, we point to EGFR/PI3K dual inhibitors as promising new molecules to treat TNBC.

## 2. Materials and Methods

### 2.1. Cell Line and Culture Conditions

The human TNBC cell line MDA-MB-231 was obtained from the American Type Culture Collection (ATCC; Manassas, VA, USA). From these parental cells, a cell line with brain tropism, green fluorescent protein (GFP)-tagged, was developed. Briefly, GFP/Luciferase (Luc) positive cells were intracardially injected in NSG mice (Charles River Laboratories, Wilmington, MA, USA) and collected once brain metastases were established. After sorting the dissociated cells, they were cultured and intracardially re-injected in mice to generate cell lines with brain tropism (Br). The cells obtained after four passages were used (MDA-MB-231 Br4). These cells were grown in Dulbecco’s modified Eagle’s medium (DMEM high glucose, #41966052, Gibco, Life Technologies, New York, NY, USA) supplemented with 10% (*v*/*v*) fetal bovine serum (FBS, Sigma Aldrich, St. Louis, MO, USA), 2 mM L-glutamine (Biochrom AG, Berlin, Germany), and 1% (*v*/*v*) antibiotic-antimycotic solution (Sigma Aldrich). The cells were maintained at 37 °C in a humid atmosphere enriched with 5% CO_2_. For experiments, MDA-MB-231 Br4 cells were seeded using a volume of 200 µL at a density of 2.5 × 10^4^ cells/mL [cell viability assay and half maximal inhibitory concentration (IC_50_) determination] in 96-well plates, or 500 µL at a density of 5.0 × 10^5^ or 5.0 × 10^4^ cells/mL in 24-well plates (wound-healing assay or immunocytochemistry, respectively). Incubation timepoints were assay-dependent.

### 2.2. Small Molecules Preparation

Twenty-seven small molecules with the predicted ability to transpose the BBB and to inhibit EGFR (molecules 1–18), PI3K (molecules 19–24), or both targets (molecules 25–27) were identified in our previous in silico analysis [39]. The chemical formula, ZINC15 ID, and molecular weight of the inhibitors are indicated in Table 1. The molecules were obtained from MolPort (MolPort SIA, Riga, Latvia) and dissolved in dimethyl sulfoxide (DMSO, Sigma Aldrich) at a stock concentration of 100 mM, aliquoted, and frozen at −20 °C until use. The subsequent dilutions for cell assays were performed in DMEM on the day of each experiment.

### 2.3. Cell Viability Assay

An initial screening was made via thiazolyl blue tetrazolium (MTT) assay. Forty-eight hours post-seeding, cells were incubated with a series of different concentrations of each molecule (0.1, 1, 10, or 100 μM) diluted in DMEM or with DMEM (with 0.1% DMSO; control). After 24 h, media were removed, and DMEM containing 0.5 mg/mL of MTT (Alfa Aesar, Haverhill, MA, USA) was added to each well and incubated for 2 h at 37 °C and 5% CO_2._ Then, the supernatants were discarded, and the formed formazan crystals were solubilized with a solution of 0.04 N HCl in isopropanol (Honeywell, Charlotte, NC, USA). Absorbance values were measured using a microplate reader (Zenyth 3100, Anthos Labtec Instruments, Salzburg, Austria) at 595 nm, and results were presented as percentages relative to the control. Three independent experiments were performed in triplicate.

### 2.4. IC_50_ Determination

The IC_50_ of the most cytotoxic compounds was determined by the MTT assay by generating 7-point dose-response curves with concentrations ranging from 0.0016 to 250 μM in DMEM after 24 h incubation. IC_50_ values were calculated by fitting the data in a standard four-parameter sigmoidal dose-response curve using GraphPad Prism^®^ 8.0.2 (GraphPad Software, San Diego, CA, USA). Three independent experiments were performed in triplicate.

### 2.5. Wound-Healing Assay

The MDA-MB-231 Br4 cell migration was evaluated by a wound-healing assay, as usual [40]. Upon cell cultures confluence, a longitudinal straight scratch with a constant diameter stripe was made using a 10 μL sterile pipette tip, followed by cells washing three times with Hank’s Balanced Salt Solution (HBSS, Gibco). The cells were then incubated in the absence (0.1% DMSO in DMEM; control) or in the presence of each selected small molecule (in DMEM) at IC_50_′s concentration. Then, 24 or 48 h incubations were performed in the absence of FBS to avoid proliferation, and results are presented as percentages relative to the control condition. Three independent experiments were performed in triplicate.

### 2.6. Immunofluorescence and Immunocytochemistry

Forty-eight hours post-seeding, the cells were incubated with each molecule at the respective IC_50_′s concentration or with DMEM (with 0.1% DMSO; control) for different timepoints.

Immunocytochemistry was performed as usual in our lab [41], using the antibodies indicated in Table 2. Briefly, at the end of the incubation period, the cells were fixed with 4% (*w*/*v*) paraformaldehyde (PFA, Sigma Aldrich) solution for 20 min at room temperature, followed by cell permeabilization with 0.3% Triton X-100 (VWR International, Radnor, PA, USA) for 5 min and blocking with 3% bovine serum albumin (BSA, Sigma Aldrich) in phosphate-buffered saline (PBS), for 60 min at room temperature. After blocking, cells were probed with primary antibodies overnight at 4 °C. Thereafter, the cells were incubated with the corresponding secondary antibodies for 60 min at room temperature. Both primary and secondary antibodies were diluted in a blocking solution. Nuclei were counterstained with Hoechst dye 33342 (Thermo Fisher Scientific, Waltham, MA, USA; 20 µM) for 10 min at room temperature. Between incubations, cells were washed three times with PBS. Methanol-dehydrated cells were then mounted on microscopy slides with DPX (Merck Millipore, Burlington, MA, USA), properly dried, and stored at 4 °C until image acquisition. To validate the specificity of labeling, negative controls without primary antibodies were performed. For each protein, three independent experiments were performed.

### 2.7. Image Acquisition and Analysis

Cyclin D2, FOXP1, Ki-67, and MEF2C, as well as the nuclei and cell morphology analysis, were performed by immunofluorescence. An Olympus BX60 microscope equipped with an Olympus U-RFL-T Mercury lamp and Hamamatsu Orca R2 cooled monochromatic CCD camera, with 40× or 100× oil objectives, were used. The images obtained were examined using Icy 1.8.6.0 (Institute Pasteur and France BioImaging, Paris, France) and ImageJ 1.54d (National Institutes of Health, Bethesda, MD, USA) software. Cell and nuclei immunoreactivity were quantified using the polygon tool in Icy 1.8.6.0 software. Five cells per field and ten fields of each cell culture condition were evaluated. The plot profiles for GFP and Hoechst localization were obtained by the plot profile tool in ImageJ 1.54d software in a representative cell in each studied condition. Briefly, a line through the center of the nucleus, and connecting both ends of the cell was drawn. Then, the fluorescence intensity of GFP and Hoechst along the distance between both edges of the drawn line was plotted.

The morphological parameters (cell and nuclei area and perimeter) were measured using all cells per field (more than 150 cells/nuclei per sample) using the polygon tool in Icy 1.8.6.0 software.

In the wound-healing assay, the widefield images were captured after 0, 24, and 48 h using a 10× objective with a phase contrast microscope (Nikon ECLIPSE TS100, Jenoptik, Jena, Germany) equipped with a Nikon ELWD camera. Three independent experiments were performed in triplicate, and one image per well was acquired. The wound closure was quantified using the line tool in ImageJ 1.54d software to measure the distance from the wound edges. Three lines per image were drawn, corresponding to three different parts of the image (two field edges and the midline), and the results are presented as wound closure percentage of control (0 h) using the following equation:

$$\%\;\mathrm{closure}=100-\left(\frac{\mathrm{distance}\;\mathrm{at}\;\mathrm{chosen}\;\mathrm{timepoint}\;\times 100\;}{\mathrm{distance}\;\mathrm{at}\;0\;h}\;\right)$$

### 2.8. Statistical Analysis

Results were analyzed using GraphPad Prism^®^ 8.0.2 (GraphPad Software, San Diego, CA, USA) and are expressed as mean ± SEM. The results represent the average of three independent experiments (*n* = 3). Before each statistical analysis, the normal distribution of the values was evaluated using the D’Agostino and Pearson test. If the values presented a normal distribution, the results were analyzed using an ordinary One-way ANOVA multiple comparisons with Tukey’s correction. If not, a Kruskal–Wallis test was used. Statistically significant differences were considered when *p* < 0.05.

## 3. Results

### 3.1. Characterization of the Developed Cell Line MDA-MB-231 Br4

**Figure 1:**
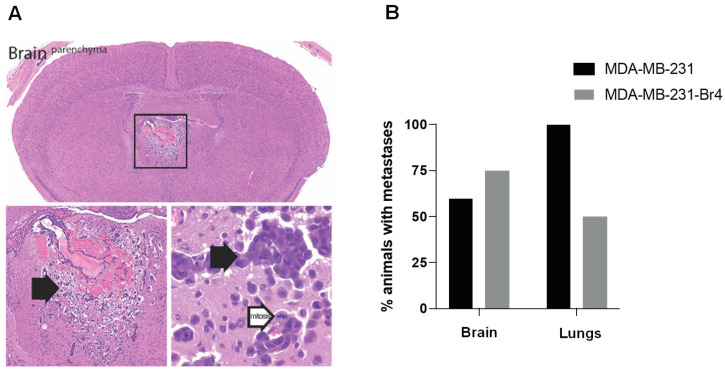
Metastatic pattern of parental breast cancer cells (MDA-MB-231) and cells with brain tropism (MDA-MB-231 Br4). NGS mice were injected in the left cardiac ventricle with MDA-MB-231 cells tagged with green fluorescent protein/luciferase (GFP/Luc). After metastases establishment, cells were collected, sorted, cultured, and intracardially re-injected. Upon four passages, the cells with brain tropism were obtained (MDA-MB-231 Br4). (**A**) A representative image of a brain section stained for hematoxylin eosin shows well-established metastases in the brain parenchyma (square and corresponding enlarged images where black arrows point to metastases and a white arrow points to a dividing cell). (**B**) Percentage of animals injected with MDA-MB-231 or MDA-MB-231 Br4 cells developing brain and lung metastases.

The pattern of dissemination of parental MDA-MB-231 and MDA-MB-231 Br4 cells was analyzed (Figure 1). As shown in Figure 1A, well-established brain metastases are observed in mice injected with MDA-MB-231 Br4 cells. The percentage of mice with brain metastases increase from 60% when parental cells were injected to 75% when the cells with brain tropism were used. As far as lung metastases are concerned, they occur in all the animals injected with MDA-MB-231 cells but only in half of those injected with MDA-MB-231 Br4. Collectively, these results show that the use of the generated cell line indeed favors the development of metastases in the brain while disfavoring the occurrence of metastases in the lungs (Figure 1B).

### 3.2. Small Molecules Cytotoxic Effects in TNBC Cells

**Figure 2:**
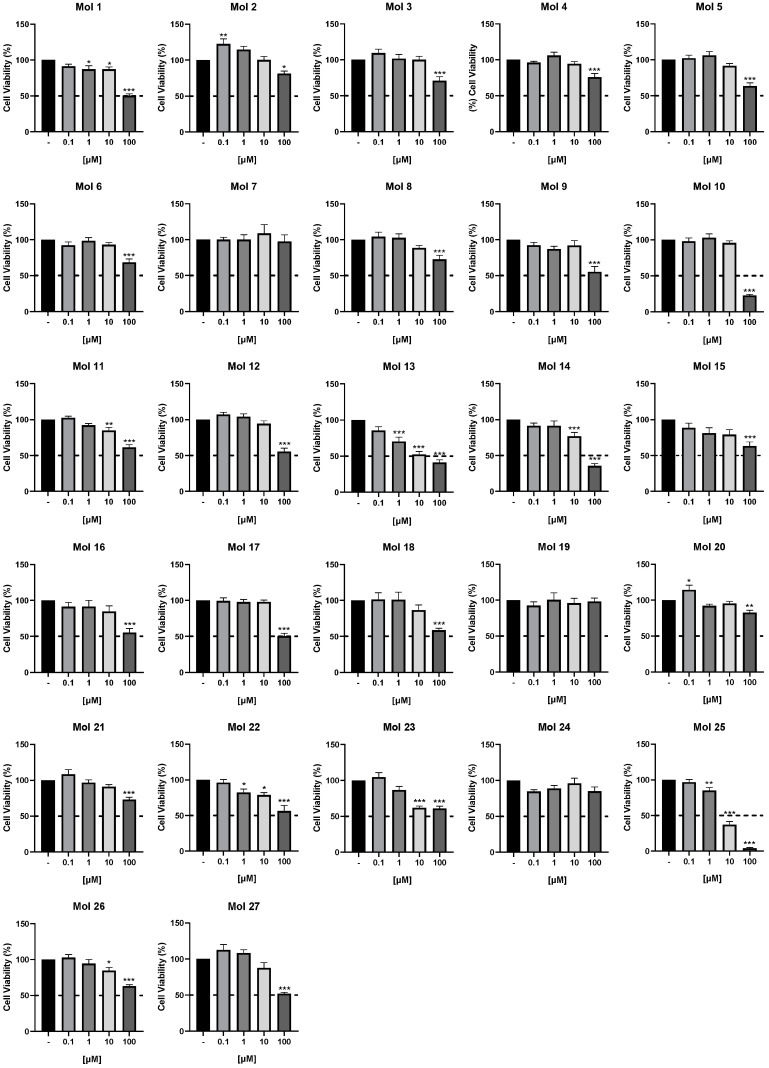
Cell viability of a highly metastatic triple-negative breast cancer cell line upon exposure to small molecule inhibitors. MDA-MB-231 Br4 cells were treated with 0.1, 1, 10, or 100 μM of epidermal growth factor receptor (EGFR) inhibitors [molecules (mol) 1–18], phosphoinositide 3-kinase (PI3K) p110β inhibitors (mol 19–24), EGFR + PI3Kp110β inhibitors (mol 25–27), or DMEM (with 0.1% DMSO; control), for 24 h. Cell viability was assessed by MTT assay, and the values are presented as percentages relative to the control. Dashed lines indicate 50% cell viability. Data are given as mean ± SEM of three independent experiments performed in triplicate. A One-way ANOVA test was used to evaluate the significant differences, represented by *** *p* < 0.001, ** *p* < 0.01, and * *p* < 0.05 vs. control.

An initial cytotoxicity screening was performed to select the most promising small molecules among the 27 inhibitors’ library, including inhibitors of EGFR (molecules 1–18), of PI3Kp110β (molecules 19–24), and of both EGFR and PI3Kp110β (molecules 25–27). To this end, cell viability was determined by the MTT assay in the human TNBC cell line with brain tropism, MDA-MB-231 Br4 cells (Figure 2).

Cells were exposed to different concentrations (0.1, 1, 10, or 100 μM) of each drug, or no addition (control), for 24 h to evaluate the cytotoxic profile of each of the inhibitors. We observed that three of the molecules (7, 19, and 24) presented no toxic effects for MDA-MB-231 Br4 cells in any of the concentrations tested. We also found that several molecules only induced toxicity at 100 µM (molecules 2, 3, 4, 5, 6, 8, 9, 10, 12, 15, 16, 17, 18, 20, 21, and 27) and that some of them (molecules 2 and 20) even promoted a significant increase in tumor cell viability at the lowest concentration tested (0.1 μM). In contrast, others were more potent, inducing a significant loss of viability at 1 µM (molecules 1, 13, 22, and 25), mostly presenting a dose-response profile. The inhibitors for further study were selected based on the following criteria: (1) inclusion of at least two molecules of each of the different groups (i.e., PI3K inhibitor, EGFR inhibitor, and dual inhibitor); (2) inclusion of those inducing at least 50% decrease in cell viability upon exposure to the highest concentration of the drug; or (3) inclusion of drugs promoting a dose-dependent viability impairment with significant effects already observed with 1 µM. As a result, three EGFR inhibitors (molecules 10, 13, and 14), two PI3Kp110β inhibitors (molecules 22 and 23), and two dual inhibitors (molecules 25 and 27) were selected.

### 3.3. Determination of Half-Maximal Inhibitory Concentration of Inhibitors

**Figure 3:**
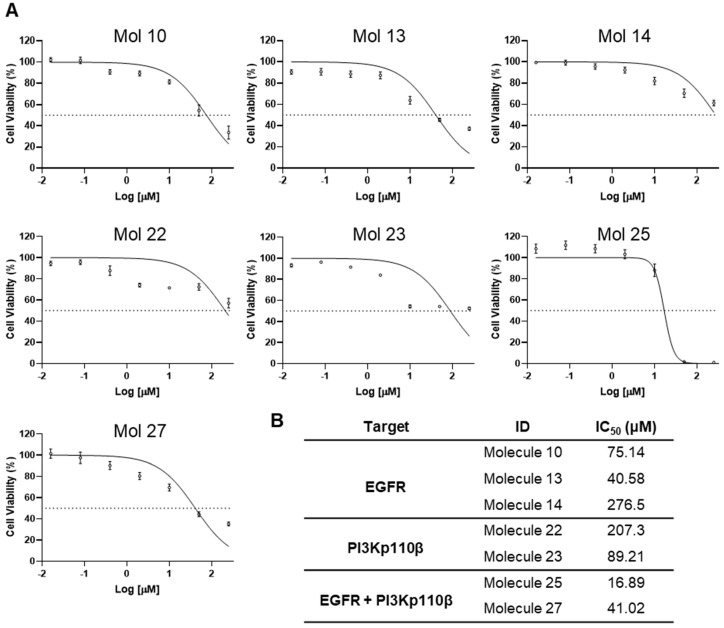
Drug-response curves delivered IC_50_ values for small molecule inhibitors. MDA-MB-231 Br4 cells were treated with seven different concentrations, ranging from 0.016 to 250 μM, of epidermal growth factor receptor (EGFR) inhibitors [molecules (mol) 10, 13, or 14], phosphoinositide 3-kinase (PI3K) p110β inhibitors (mol 22, 23); and dual inhibitors (mol 25, 27) for 24 h, and cell viability was assessed by the MTT assay and results were expressed as percentages from the control. (**A**) Graphical representation of the cytotoxic profile of each mol, with cell viability in percentage (%) vs. concentration (μM). Data are given as mean ± SEM of three independent experiments performed in triplicate. (**B**) IC_50_ values obtained for each mol from a four-parameter sigmoidal dose-response curve.

Having selected the top seven small molecules with significant toxicity in MDA-MB-231 Br4 cells, we aimed to determine their IC_50_ (Figure 3) to be used in the following experiments.

Dose-response curves for concentrations ranging from 0.016 to 250 μM were determined after 24 h incubation with each of the selected molecules (Figure 3A). A standard four-parameter sigmoidal dose-response curve was obtained, and the IC_50_ value was calculated (Figure 3B). We observe that molecule 25 presents the lowest IC_50_ value, followed by molecule 13, molecule 27, and lastly, by molecules 10 and 23, which present values of IC_50_ below 100 µM. On the other hand, molecules 14 and 22 present the highest IC_50_ values, above 200 μM, and for that reason, were excluded from the following experiments.

### 3.4. Small Molecule Inhibitors Induce TNBC Cell Death

**Figure 4:**
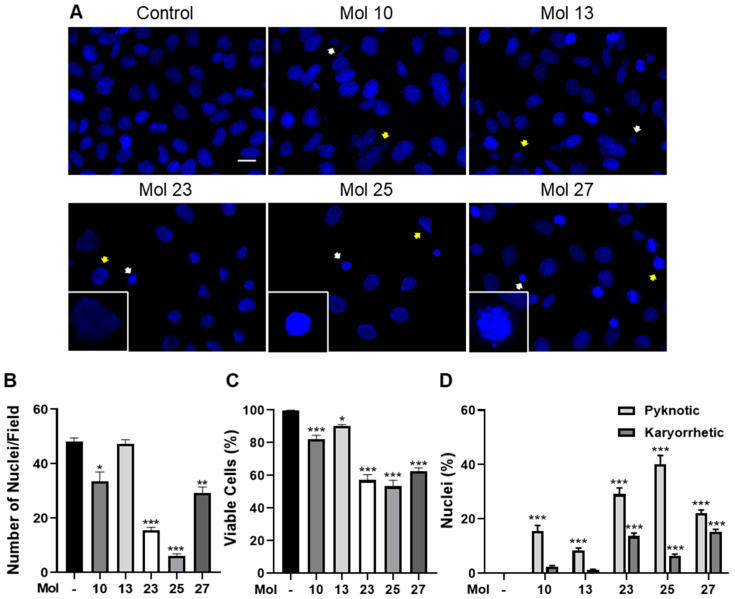
Small molecule inhibitors induce highly metastatic triple-negative breast cancer cells death. MDA-MB-231 Br4 cells were incubated with molecule (mol) 10, 13, 23, 25, or 27 at IC_50_′s concentration, or DMEM (with 0.1% DMSO; control), for 24 h, and nuclei number and morphological alterations were evaluated following nuclei labeling with Hoechst 33342 (blue). (**A**) Analysis of immunofluorescence images revealed a decrease in nuclei number in the presence of inhibitors, as well as an increase of pyknotic (white arrows) and karyorrhectic (yellow arrows) nuclei. Insets highlight major alterations magnified. Scale bar: 20 µm. Semi-quantitative analysis showed a (**B**) decrease in the number of nuclei per field and a (**C**) decrease in the percentage of viable cells. Non-viable cells were characterized by the increase of (**D**) pyknotic and karyorrhectic nuclei. Data are given as mean ± SEM (*n* = 3, all cells/field, 10 fields/condition). A Kruskal–Wallis test was used to evaluate the significant differences, represented by *** *p* < 0.001, ** *p* < 0.01, and * *p* < 0.05 vs. control.

Due to the strong cytotoxic potential of molecules 10, 13, 23, 25, and 27, we aimed to elucidate their effect on mechanisms of programmed and non-programmed cell death (apoptosis and necrosis, respectively) in TNBC cells, a usual downstream effect of EGFR/PI3K inhibition [42,43]. For that, MDA-MB-231 Br4 cells were treated with each of the five selected molecules in their IC_50_ concentration for 24 h, and nuclei were labeled with Hoechst for immunofluorescence analysis (Figure 4).

Exposure to the inhibitors led to alterations in nuclei number, reflecting cell detachment as a result of increased cell death. Moreover, analysis of nuclei morphology revealed an increase in pyknosis (shrinkage nuclei morphology) and karyorrhexis (nuclei blebbed, fragmented, or with crescent morphology), morphological changes typical of necrosis and apoptosis, where chromatin condensation and nuclear fragmentation are observed (Figure 4A). Quantification of nuclei number per field after treatment with the drugs revealed that molecules 23 and 25 induced the most significant decreases (*p* < 0.001), with less than 20 nuclei/field, followed by molecule 27 (*p* < 0.01) and molecule 10 (*p* < 0.05), whereas no significant effect was observed for molecule 13 (Figure 4B). Once nuclei morphological alterations reflect cell impairment, an in-depth analysis was performed distinguishing cells with a normal nuclear morphology, considered viable, and those with aberrant nuclei, considered non-viable, and the viable cells percentage was calculated. We observed that molecules 23, 25, and 27 present the most significative reduction of viable cells, which represents 55–60% of the control (*p* < 0.001, Figure 4C). Regarding non-viable cells, quantification of pyknotic and karyorrhectic cells per field was further performed (Figure 4D). Even though presenting fewer cells per field, we observe that molecule 25 presents the highest percentage of pyknotic cells (about 40%, *p* < 0.001), followed by molecules 23 and 27 (*p* < 0.001). Interestingly, a different trend is observed regarding karyorrhectic nuclei, with molecules 23 and 27 leading to the highest increases in those nuclei (above 10%, *p* < 0.001), pointing to putative different mechanisms of cell death promotion or aggressiveness in TNBC cells by the different inhibitors tested.

### 3.5. Small Molecule Inhibitors Affect TNBC Cells Migration

**Figure 5:**
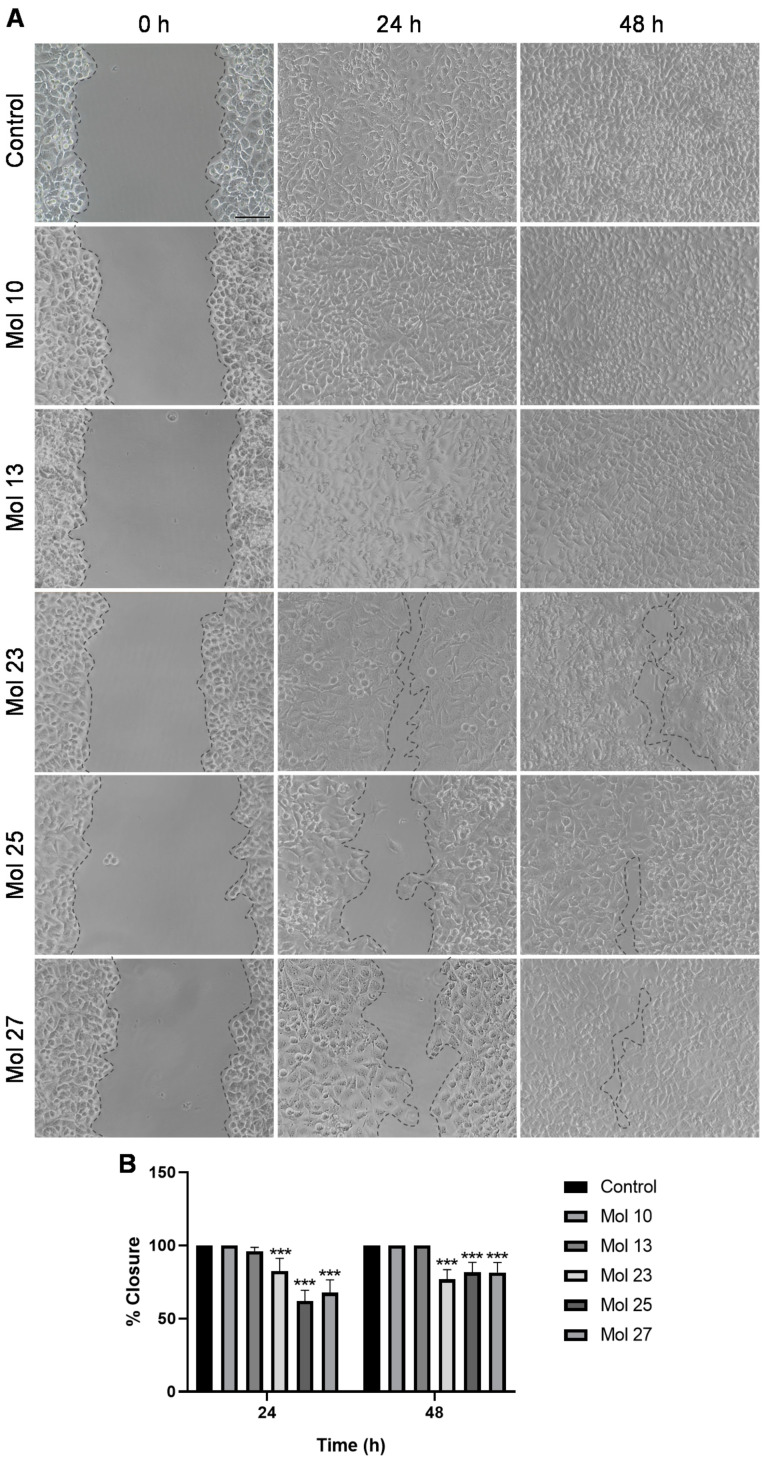
Molecules 23, 25, and 27 efficiently modulate the highly metastatic triple-negative breast cancer cell migration. Confluent monolayers of MDA-MB-231 Br4 cells were wounded and incubated with molecules (mol) 10, 13, 23, 25, or 27 at IC_50_′s concentration, or DMEM (with 0.1% DMSO; control), for 24 and 48 h. (**A**) Images of wound healing assay revealed an inhibitory effect on cell migration for mol 23 and particularly notable for mol 25 and 27 at 24 h. Scale bar: 300 µm. (**B**) Semi-quantitative analysis of the percentage of wound closure relative to the control cells validated the qualitative observations. Data are given as mean ± SEM (*n* = 3, performed in triplicate). A One-way ANOVA test was used to evaluate the significant differences, represented by *** *p* < 0.001 vs. control of each timepoint.

In order to clarify the potential of the small molecule inhibitors to impair tumor cells’ migration capacity, a key mechanism involved in tumorigenesis and downstream of EGFR/PI3K signaling [44], MDA-MB-231 Br4 cells were incubated with each inhibitor at its IC_50_′s concentration, and cell migration/wound closure was monitored 24 and 48 h after scratch (Figure 5).

Treatment of MDA-MB-231 Br4 cells with each of the EGFR inhibitors (molecules 10 and 13) does not affect cell migration. On the other hand, the PI3K inhibitor (mol 23) and, mostly, the dual-target inhibitors (molecules 25 and 27) reduce tumor cell migration and, consequently, wound closure, particularly at 24 h (Figure 5A).

Semi-quantitative evaluation of distance migrated by tumor cells (Figure 5B) reveals that molecules 25 and 27 present the lowest percentage of wound closure after 24 h (62% and 67%, respectively, *p* < 0.001), followed by molecule 23, with a wound closure percentage of 82% (*p* < 0.001). After 48 h, the percentage of wound closure for molecules 25 and 27 are similar, both at 81% (*p* < 0.001), whereas molecule 23 presents 76% of wound closure (*p* < 0.001).

In sum, combining the alterations observed regarding cell death (Figure 4) and the potential in modulating tumor cell migration (Figure 5), together with the low IC_50_ (Figure 3), we can point to molecules 25 and 27 as the best candidates for in-depth analysis of their anti-tumor mechanism of action against TNBC.

### 3.6. Dual-Inhibitor Molecules Modulate Cell Cycle and TNBC Cells Proliferation

**Figure 6:**
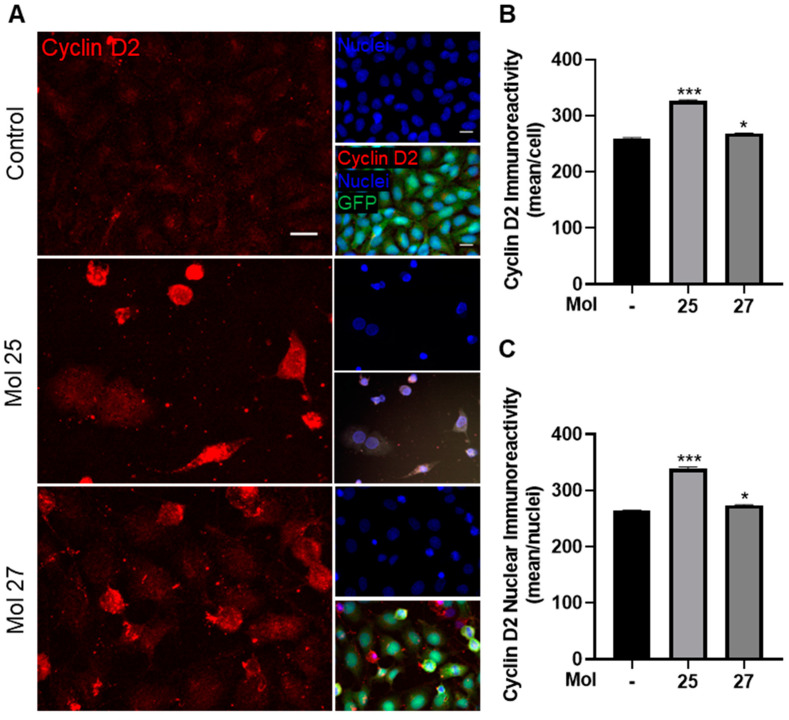
Cyclin D2 expression increases in highly metastatic triple-negative breast cancer cells after dual-inhibitor treatment. MDA-MB-231 Br4 cells [green fluorescent protein (GFP)-fused, green] were incubated with molecule (mol) 25 or 27 at IC_50_′s concentration, or DMEM (with 0.1% DMSO; control) for 24 h, and the expression of the cell cycle marker, cyclin D2 (red), was evaluated by immunofluorescence. Hoechst 33342 was used as counterstaining for nuclei (blue). (**A**) Analysis of the expression of cyclin D2 revealed an increased expression in MDA-MB-231 Br4 cells after incubation with mol 25 or 27. Scale bar: 20 µm. Cyclin D2 expression profile was quantified as the mean immunoreactivity (**B**) per cell and (**C**) per nuclei, which revealed a significant increase of the protein expression, particularly notable in the presence of mol 25. Data are given as mean ± SEM (*n* = 3, 5 cells or nuclei/field, 10 fields/condition). A Kruskal–Wallis test was used to evaluate the significant differences, represented by *** *p* < 0.001 and * *p* < 0.05 vs. control.

The effect of the molecules 25 and 27 in cell cycle progression, a key event downstream of EGFR/PI3K pathway [45], was assessed by immunocytochemistry analysis of cyclin D2 (Figure 6), a protein involved in cell cycle progression and described to be downregulated in BC [31,46].

Regarding the effects of the molecules in cyclin D2 immunoreactivity, we observed that untreated cells presented low expression of this regulator of G1–S transition, while with both molecules’ treatment, an increase was observed (Figure 6A).

Semi-quantitative analysis (Figure 6B,C) shows stimulation of cyclin D2 expression by each of the molecules, with molecule 25 presenting the highest immunoreactivity all over the cell and in the nuclei (*p* < 0.001 and *p* < 0.05 vs. control group for molecules 25 and 27, respectively). Overexpression of cyclin D2 is concomitant with the shrinkage of the nuclei, as noticed in the microscopy images (Figure 6A), indicating that the drug-induced cell death is at least partially mediated by cyclin D2.

**Figure 7:**
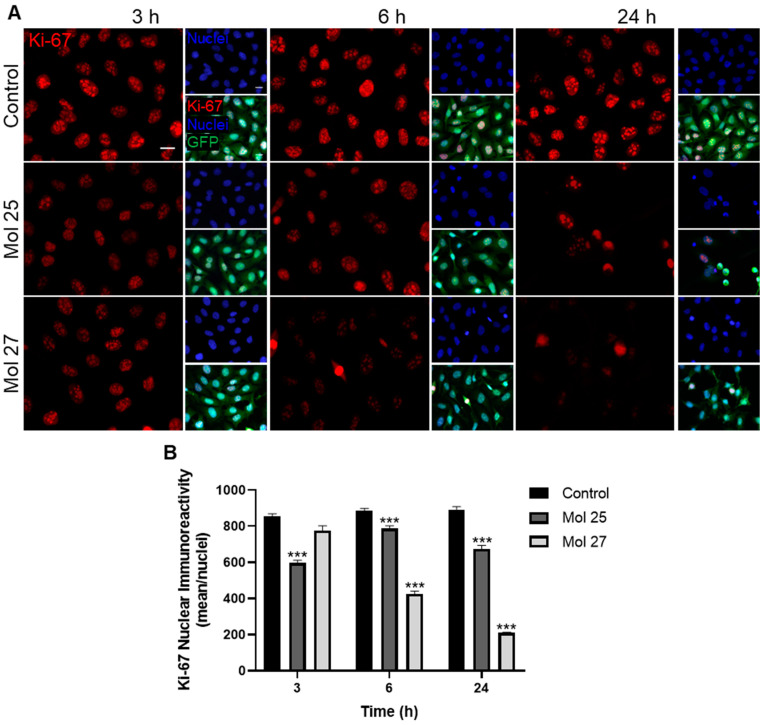
Dual inhibitors impair highly metastatic triple-negative breast cancer cell proliferation. MDA-MB-231 Br4 cells [green fluorescent protein (GFP)-fused, green] were incubated with molecule (mol) 25 or 27 at IC_50_′s concentration, or DMEM (with 0.1% DMSO; control) for 3, 6, and 24 h, and the expression of the proliferation marker, Ki-67 (red), was evaluated by immunofluorescence analysis. Hoechst 33342 was used as counterstaining for nuclei (blue). (**A**) Analysis of the nuclear expression of Ki-67 showed a decrease in protein expression after the treatment with mol 25 and 27. Scale bar: 20 µm. Semi-quantitative analysis of Ki-67 nuclear expression (**B**) revealed a significant decrease of Ki-67 nuclear immunoreactivity after incubation with mol 25 or 27 from 3 and 6 h onwards, respectively. Data are given as mean ± SEM (*n* = 3, 5 nuclei/field, 10 fields/condition). A Kruskal–Wallis test was used to evaluate the significant differences, represented by *** *p* < 0.001 vs. control of each timepoint.

To strengthen the observations of the dual inhibitors towards the cell cycle mediated by cyclin D2 overexpression, we further assessed the cell proliferation status. To this end, we analyzed the temporal expression of Ki-67, a key player involved in tumor cell proliferation [47], in the presence of molecules 25 or 27 at their IC_50_′s concentration (Figure 7).

Immunocytochemistry analysis demonstrates a Ki-67 typical nuclear expression in MDA-MB-231 Br4 cells, which show a decrease starting at 3 h treatment, with a more noticeable decrease along time observed for molecule 27 (Figure 7A). The semi-quantitative analysis of the proliferation marker expression in cells treated with molecule 25 reveals a sustained decrease at 6 and 24 h (*p* < 0.001 vs. control at each timepoint, Figure 7B). As far as molecule 27 is concerned, a progressive and marked decrease along time is observed at 6 and 24 h, achieving ~50% and ~25% of the control immunostaining, respectively (*p* < 0.001, Figure 7B), pointing to a clear role of this molecule in reducing tumor cells proliferation.

Collectively, we can conclude that molecule 27 presents a higher effect than molecule 25 in modulating tumor cell proliferation, though molecule 25 still possesses a strong effect against TNBC cells despite almost all cells being completely damaged at 24 h.

### 3.7. Dual-Inhibitor Molecules Alter TNBC Cells Morphology

**Figure 8:**
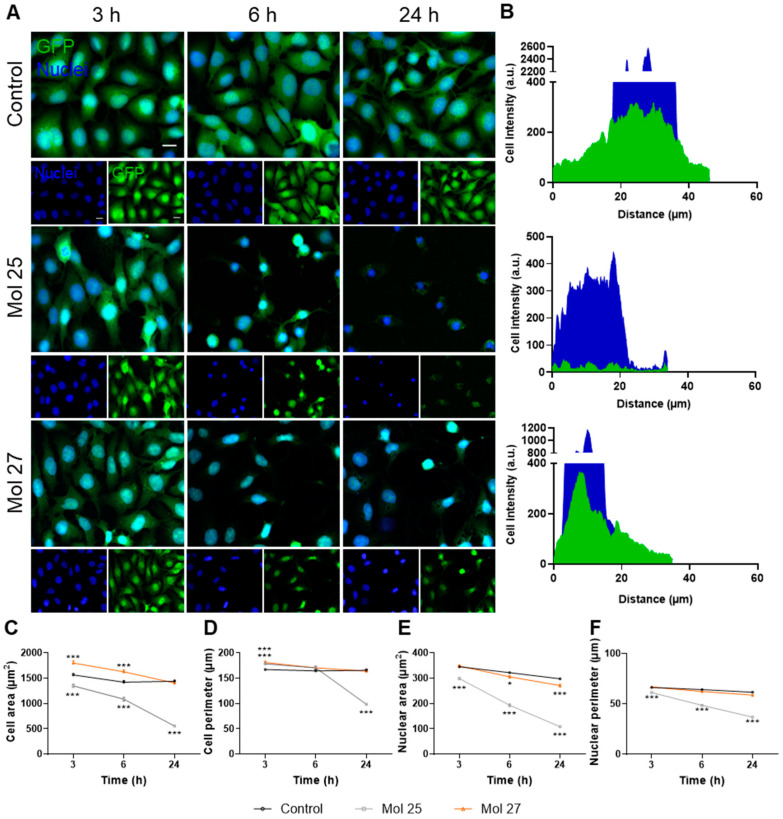
Dual inhibitors modulate highly metastatic triple-negative breast cancer cell morphology. MDA-MB-231 Br4 cells [green fluorescent protein (GFP)-fused, green] were incubated with molecule (mol) 25 or 27 at IC_50_′s concentration or DMEM (with 0.1% DMSO; control) for 3, 6, and 24 h and the cell morphology was evaluated by fluorescence analysis. Hoechst 33342 was used as counterstaining for nuclei (blue). (**A**) Fluorescence images showed that mol 25 and 27 promoted morphological changes after 3 h, with an increase in cell shrinkage and nuclear condensation, particularly notable after 6 h. Scale bar: 20 µm. (**B**) The localization of green and blue labeling (corresponding to the GFP constitutively expressed by cells and the cell nuclei, respectively) at 24 h was studied using a plot profile of fluorescence intensity throughout a malignant cell (edge to edge), which confirmed the dramatic morphological alterations in MDA-MB-231 Br cells, mainly after the treatment with mol 25. The characterization of morphological alterations in MDA-MB-231 Br4 cells over time was performed by the quantification of the cell (**C**) area and (**D**) perimeter, as well as of the nuclear (**E**) area and (**F**) perimeter, which decreased along time, particularly for mol 25. Data are given as mean ± SEM (*n* = 3, all cells/field, 10 fields/condition, corresponding to more than 150 cells/condition). A One-way ANOVA test was used to evaluate the significant differences, represented by *** *p* < 0.001 and * *p* < 0.05 vs. control of each timepoint.

Profiting from the fact that MDA-MB-231 Br4 cells are green fluorescent protein (GFP)-positive, representative images of GFP channel and nuclei stained with Hoechst 33342 were acquired after 3, 6, and 24 h, and alterations in cell and nuclear morphology were analyzed to evaluate the effect of each dual inhibitor in cells phenotype along time (Figure 8).

We observed that both molecules have effects in tumor cells as early as at 3 h, where some morphological changes were already spotted, namely in GFP signal and nuclear chromatin condensation (Figure 8A). At 6 h, a significant decrease in malignant cells per field is observed in the presence of each of the two tested molecules, while the remaining cells present more nuclear GFP, being more evident in molecule 25 (Figure 8A). At 24 h, it is clear that the cells incubated with molecule 25 are destroyed, with barely any GFP staining, showing cytoplasm shrinkage and loss of plasma membrane integrity (Figure 8B).

Semi-quantitative analysis of morphological alterations highlights a pronounced decrease of cell area with molecule 25 (*p* < 0.001 at 3, 6, and 24 h vs. control at each timepoint, Figure 8C). Surprisingly, we also observed an increase in cell area with molecule 27 (*p* < 0.001 at 3 and 6 h vs. control at each timepoint, Figure 8C), which decreases after 24 h to values comparable to untreated cells. Additionally, the most marked decrease in cell perimeter is observed with molecule 25 at 24 h (*p* < 0.001, Figure 8D). Moreover, alterations regarding the nuclear area and perimeter are observed, with a dramatic decrease along time observed for molecule 25 (*p* < 0.001 vs. control at each timepoint, Figure 8E,F). These findings support the previous observations regarding nuclei shrinkage, which are more notable for mol 25 than for mol 27.

Taking into consideration all the parameters evaluated, it is noticeable that molecule 25 presents a stronger and earlier effect compared with molecule 27, obliterating almost all the cells at 24 h, while molecule 27 still has a strong effect in these highly metastatic cells, but cell integrity is maintained longer in time. Due to the serious injury observed from 6 h onwards, with marked cell shrinkage and nuclear condensation, subsequent studies on signaling molecules were performed at 3 h.

### 3.8. Dual-Inhibitor Molecules Have Downstream Effects in EGFR/PI3K Pathway

**Figure 9:**
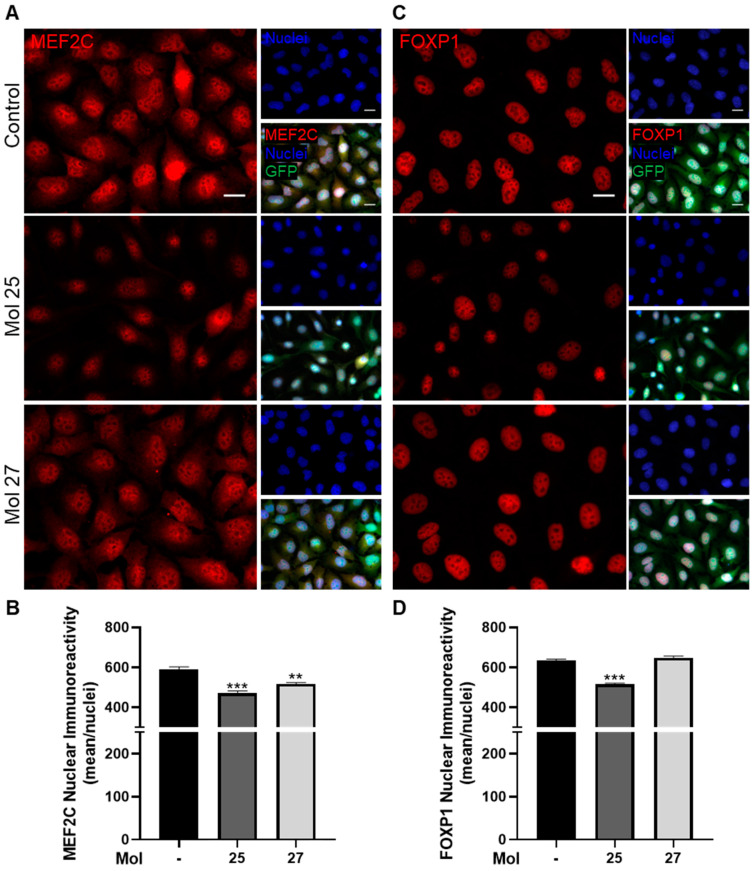
Myocyte enhancer factor 2C (MEF2C) and forkhead box P1 (FOXP1) expression in triple-negative breast cancer cells are affected by dual-inhibitor treatment. MDA-MB-231 Br4 cells [green fluorescent protein (GFP)-fused, green] were incubated with molecule (mol) 25 or 27 at IC50′s concentration, or DMEM (with 0.1% DMSO; control) for 3 h, and the expression of MEF2C (red) and FOXP1 (red) was evaluated by immunofluorescence analysis. Hoechst 33342 was used as counterstaining for nuclei (blue). (**A**) Analysis of MEF2C immunoreactivity showed a decreased expression upon treatment. Scale bar: 20 µm. Semi-quantitative analysis of nuclei MEF2C immunoreactivity per cell (**B**) highlighted an inhibitory effect of both mol 25 and 27 in tumoral MEF2C expression. (**C**) Analysis of FOXP1 immunoreactivity indicated a decreased expression with mol 25 treatment. Scale bar: 20 µm. (**D**) Semi-quantitative analysis of FOXP1 nuclear mean immunoreactivity validated a decreased expression of FOXP1 in tumor cells treated with mol 25. Data are given as mean ± SEM (*n* = 3, 5 nuclei/field, 10 fields/condition). A Kruskal–Wallis test was used to evaluate the significant differences, represented by *** *p* < 0.001 and ** *p* < 0.01 vs. control.

To evaluate the effect of each molecule in downstream EGFR/PI3K pathway markers important for cell survival and migration, the expression of MEF2C and FOXP1, and particularly the nuclei localization of these transcription factors, were assessed by immunocytochemistry (Figure 9). MDA-MB-231 Br4 cells were subjected to each molecule at its IC_50_′s concentration for 3 h.

MEF2C, involved in migration/invasion and proliferation of TNBC cells and associated with an aggressive phenotype of BC brain metastasis [37,38], was also evaluated (Figure 9A,B). A clear basal MEF2C presence is observed in MDA-MB-231 Br4 cells, especially in the cell nuclei, which were modulated in the presence of the dual inhibitors (Figure 9A). The semi-quantitative analysis reflects the observed changes in the pattern of nuclear MEF2C immunoreactivity. In fact, both molecules promote a significant decrease in MEF2C in the nuclei (*p* < 0.001 and *p* < 0.01 from control for molecules 25 and 27, respectively, Figure 9B), supporting their inhibitory potential in the downstream pathway, which is in line with our previous observations [37,38].

The significance of FOXP1, an important transcription factor related to the migration of malignant cells [48], was also analyzed (Figure 9C,D). Immunocytochemistry analysis of nuclear FOXP1 immunoreactivity reveals alterations in the presence of molecule 25, unlike molecule 27, for which no alteration is noticed (Figure 9C). Semi-quantitatively, we noticed that after the treatment with molecule 25, a decrease of FOXP1 immunoreactivity compared with the control was observed (*p* < 0.001, Figure 9D), pointing to a role of this molecule in controlling tumor cells’ migration via FOXP1. On the other hand, FOXP1 immunoreactivity does not suffer significant alterations in tumor cells treated with molecule 27 (Figure 9D). These observations indicate that the anti-tumor effect of molecule 27 is not mediated by FOXP1, in contrast to the action of molecule 25.

Collectively, both modulators revealed an efficient effect in the players involved in tumorigenesis, such as MEF2C and FOXP1.

## 4. Discussion

The lack of specific targets for TNBC renders the search for an effective treatment for this BC subtype challenging, driving the research to find new ones namely by targeting EGFR, which is overexpressed in metastatic TNBC [9,49]. Unfortunately, anti-EGFR treatments have shown toxicity and low efficacy due to the coactivation and/or amplification of compensatory signaling pathways [50,51]. This makes necessary the search for novel molecules with the capacity to simultaneously tackle downstream cascade activation, such as PI3K. On the other hand, the main cause of death in patients with BC is metastases development [52], a condition that is particularly severe when the disease spreads into the brain [7]. So, we aimed to disclose EGFR, PI3K, and dual-target inhibitors to act in highly metastatic human TNBC cells with brain tropism as promising candidates for a novel therapeutic strategy to abrogate metastases. To this end, we performed a screening of a set of compounds with predicted ability to inhibit EGFR, PI3K, or both. The results obtained allowed the identification of two dual target molecules as the most promising ones, molecules 25 and 27. Importantly, the dual-targeting properties of these molecules point to their potential to avoid cell resistance to treatment due to the activation of compensatory mechanisms.

Our previous studies revealed molecules 25 and 27 as strong candidates to treat glioblastoma based on their action in two glioblastoma cell lines (U87MG and U87MG overexpressing EGFR) [39]. Interestingly, the IC50′ value obtained in U87MG cells for molecules 25 and 27 were 16.34 and 22.74 µM, which are within the same range as those obtained for the MDA-MB-231 Br4 cell line, of 16.89 and 41.02 µM, respectively, which provides support for the anti-tumor activity of these dual-target inhibitors. Molecule 25, known as canertinib or CI-1033, is an irreversible tyrosine kinase inhibitor. This molecule was previously reported to inhibit cell growth and induce apoptosis in tumor cells [53,54,55]. Moreover, preclinical studies in which canertinib was orally administered showed an efficacious delay in the growth of several tumor xenografts mice, including BC [56,57]. Phase I clinical trials were performed in patients with advanced solid malignancies, which showed the drug’s ability to downregulate EGFR and Ki-67 [58]. However, phase II trials showed modest effects in BC and non-small-cell lung carcinoma patients, which might be explained by the 31% bioavailability [57,59]. This raises the interest in developing strategies to improve the bioavailability of this promising molecule. Importantly, recent studies revealed the ability of canertinib to reverse tamoxifen resistance in BC cells, providing a rationale for its clinical evaluation in combination with other cytotoxic drugs. Interestingly, such a finding was obtained with a canertinib concentration of 10 µM [54], which is within the range used in the present study (16.89 µM). In what concerns molecule 27, it is a new dual inhibitor of EGFR and PI3Kp110β. Therefore, its potential as a drug candidate has never been reported, apart from our own in silico predictions and subsequent biological validation in glioma cell lines. Interestingly, glioma cells responded differently to molecules 25 and 27 [39], corroborating the different behavior of TNBC cells observed in the present study.

The present results highlight that molecule 25 has the greatest effect on cell death promotion, leading to the formation of more pyknotic nuclei compared with molecules 23 and 27, which have a greater prevalence of fragmented, swelled, ruptured, or blebbed nuclei. Therefore, this different behavior points to different mechanisms of cell death. In fact, after 24 h, molecule 25 led to cell shrinkage and chromatin condensation. In turn, molecule 27 induced a reduction in viable cells and morphological parameters, not glaring as molecule 25. Interestingly, the greater potency of molecule 25 as compared with molecule 27 was also observed in glioma cells [39], indicating that the cytotoxicity is independent of the malignant cell type.

**Figure 10:**
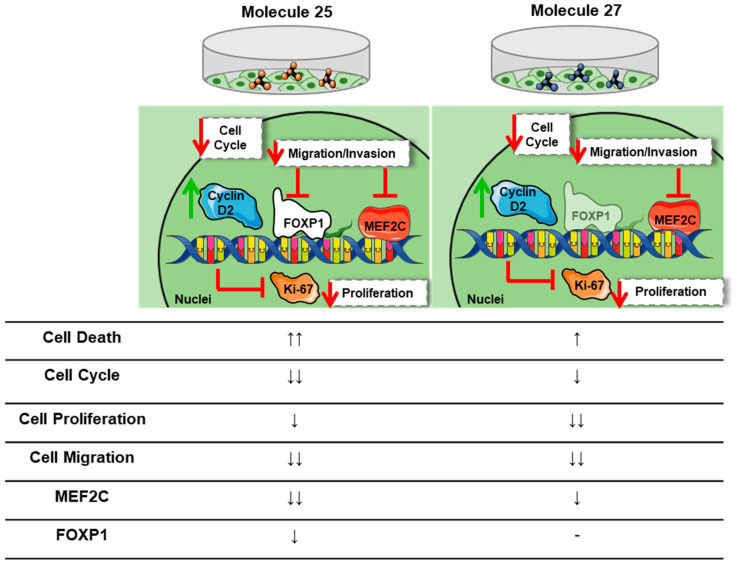
Summary of the effects of molecule (mol) 25 and mol 27 in highly metastatic triple-negative breast cancer (TNBC) cells. The exposure of TNBC cells to mol 25 promoted the expression and activation of cyclin D2, in line with cell cycle inhibition. Moreover, mol 25 inhibited Ki-67 and forkhead box P1 (FOXP1) expression at the cell nuclei, decreasing the TNBC cell’s proliferation and migration abilities. An inhibitory effect in cytoplasmic myocyte enhancer factor (MEF)2C expression was also observed, pointing to a decrease in cellular processes such as cell invasion. On the other hand, mol 27 presented inhibitory effects towards Ki-67 and MEF2C, mainly affecting invasive/migratory and proliferative properties of malignant cells, with the cell cycle appearing to be inhibited, as observed by the increased expression of cyclin D2. Together, both mol 25 and 27 appear as strong candidates to tackle TNBC. Arrows indicate the inducer (up) or inhibitor (down) potential of each mol in the cellular events; the number of arrows increases with the stronger effect of mol 25 and 27. (-) indicates that no effect was observed or conclusive.

Mechanisms of apoptosis and/or necrosis have been associated with the appearance of pyknotic cells/nuclei in BC cells treated with the EGFR inhibitor erlotinib or with jolkinolide B, which targets the PI3K/AKT pathway [60,61]. To note that phosphorylation of specific proteins downstream to AKT is needed for cell shape changes that precede migration, such as the formation of extended membrane protrusions [62]. Previous studies have established that cancer cells have a less sturdy and more pliable nuclear envelope compared with non-neoplastic cells, a consequence of the loss/reduction of proteins that maintain the nuclei structure stability, such as lamin A/C [63]. Such alterations have been simultaneously considered a weakness for the tumor cells, but it also opens doors for cancer treatment. Paclitaxel, a widely used chemotherapeutic agent, has been described to act by mitotic and non-mitotic mechanisms. Via non-mitotic mechanisms, paclitaxel may enter the cell’s nuclei, inducing the formation of rigid microtubules and promoting nuclei fragmentation (multimicronucleation) in an independent way of mitosis phenomena [64]. Our results for molecules 23 and 27, where a clear nuclei fragmentation on the tumor cells is observed, seem to indicate a similar mechanism of action to clinically approved drugs, such as paclitaxel. On the other hand, molecule 25 appears to produce a different effect in BC cells, characterized by the condensation of nuclei chromatin and decreased size/diameter, resembling a pyknosis mechanism. Altogether, we can argue that the selected drug candidates appear to act by distinct mechanisms (Figure 10), a difference already observed by us when testing these same molecules in glioblastoma [39]. Therefore, the induced morphological nuclear alterations may underly distinct drugs’ effect in preventing mechanisms beyond cell division, such as cell migration.

Cell invasion is a crucial aspect in metastases development [65]. Thus, the ability of the top five molecules (10, 13, 23, 25, and 27) to modulate migration was ascertained by a wound-healing assay. We observed that molecules 23, 25, and 27 had the highest capacity to inhibit tumor cell migration. Previous studies with canertinib in a 3-dimensional culture model that recapitulates the stromal environment of BC also showed its potential to reduce cell migration distance and the number of migrated BC cells [66]. Interestingly, several molecules extracted from plants that target PI3K have shown effects in TNBC cells’ migration, such as salidroside (40 μM), platycodin D (15 μM), and naphtho[1,2-b]furan-4,5-dione (3 μM) [67,68,69]. Our results, using molecules in the same range of concentrations, are encouraging, reinforcing the potential of targeted therapy to modulate tumor cell migration.

Closely related to the EGFR/PI3K signaling pathway in cancer are mechanisms of cell cycle and proliferation regulation [70,71]. Cyclin D2 is a protein involved in the cell cycle progression, found to be downregulated in TNBC due to promoter hypermethylation [31,32]. Moreover, an increase in cyclin D2 is associated with tumor-suppressing behavior, inhibiting tumor cell growth and leading to apoptosis [33,72]. We found no relevant expression of cyclin D2 in MDA-MB-231 Br4 cells, in line with the previous reports of lack of expression in cultured BC cells and in breast adenocarcinoma tissue [31,32,33]. Upon exposure to molecules 25 and 27, and particularly to molecule 25, an increased cyclin D2 immunoreactivity was observed, indicating that the anti-tumor activity of these drugs involves cell cycle inhibition. In line with this observation, a natural product known as antroquinonol D, which has the PI3K as one of its targets, was shown to induce the gene encoding for cyclin D2, *CCND2*, DNA demethylation, recovering the *CCND2* expression and leading to the inhibition of TNBC cell growth [33]. Moreover, a synthetic inhibitor of histone deacetylases inhibited cell proliferation attributed to cell cycle arrest by modulation of cell cycle genes, including the tumor suppressor gene cyclin D2 [72].

To assess cell proliferation, we analyzed Ki-67, a protein expressed in the nucleus of the cells during different phases of the cell cycle. High levels of Ki-67 expression in BC strongly correlated with worse survival, poor prognosis, and aggressive clinical feature [73,74]. The reduction of Ki-67 expression by the dual inhibitors, with molecule 25 having an earlier effect and molecule 27 presenting a more pronounced and gradual outcome over time, corroborated the efficiency of these drug candidates in counteracting the proliferative status of malignant cells. Reduction of Ki-67 was reported in TNBC tumors treated with gefitinib and with an EGFR inhibitor in combination with fingolimod, as well as in human BC cell xenograft tumors treated with platycodin D [68,75], reinforcing our conclusions of significant decreases in tumor cells proliferation.

Important markers related to malignant cell features downstream of PI3K signaling are the transcription factors MEF2C and FOXP1 [23,27]. MEF2C is highly expressed in BC and in brain metastases from BC. In fact, it was found in the cytoplasm, and when activated, it translocates into the nuclei to control the expression of proteins related to tumor cell proliferation and migration/invasion, correlating with a highly metastatic profile and advanced disease stages [36,37,38]. We observed that both molecules decreased the expression of this transcription factor in the nuclei. To the best of our knowledge, this is the first work unveiling the effect of EGFR/PI3K inhibitors on MEF2C. FOXP1 is an oncogene found to be overexpressed in BC cells, enhancing their migratory properties [27,30]. Here, we found a sustained reduction of FOXP1 nuclear expression in the presence of molecule 25. Interestingly, it has been reported that molecule 25 is able to dephosphorylate p70S6-kinase T389, an upstream regulator of FOXP1 [76], which corroborates our findings. It was also reported that the use of the PI3K inhibitor, wortmannin, promotes a decrease in FOXP1 expression, supporting the fact that PI3K inhibitors have an effect on this transcription factor [27]. Regarding the role of FOXP1 in BC progression, it has been shown that its overexpression promotes an increase in the proliferation [77] and migration of BC cells [48], supporting the pro-tumorigenic role of FOXP1 in BC. In this line, the decrease in FOXP1 immunoreactivity observed after treatment with molecule 25 can explain the inhibitory effect of this molecule on the BC cell’s properties, such as migration and proliferation. Regarding molecule 27, no alteration was noticed, having values of immunoreactivity close to the control, establishing that this molecule may not be the best modulator of this transcription factor.

Collectively, the results here presented indicate that molecules 25 and 27 impair crucial aspects of TNBC, including tumor cell invasion/migration and proliferation, and block the progression of the cell cycle by modulating multiple signaling pathways in highly metastatic cells (Figure 10). Importantly, to date, no studies were found focused on the use of such molecules towards this particular BC subtype, one of the most metastatic and with the poorest prognosis, particularly when brain metastases develop. Therefore, this work paves the way for the development of a therapy for such a devastating disease. Worth highlighting is the fact that these molecules have also shown promising results against glioblastoma cells, which further extends their potential against other types of malignancies.

## 5. Conclusions

The studies here reported, relying on an experimental screening of small molecules with the computationally predicted ability to inhibit EGFR, PI3K, or both, revealed two dual-target inhibitors as the most promising drug candidates to counteract TNBC tumorigenesis using a highly metastatic cell line with brain tropism. Moreover, the results disclosed the drugs’ efficacy to induce cell death, interfere with the cell cycle, and abrogate proliferative and invasive features of malignant cells. They also highlighted the modulatory effects on the tumor-associated transcription factors MEF2C and FOXP1. Overall, both molecules represent strong candidates for TNBC treatment, with impressive anti-tumor effects in low-range doses and in short exposure times.

## Figures and Tables

**Table 1:** List of the used small molecule inhibitors according to their target, molecule ID, chemical formula, ZINC15 database ID, and molecular weight.

Target	Molecule (Mol) ID	Chemical Formula	ZINC ID	Molecular Weight (g/mol)
EGFR	1	C_14_H_10_BrN_3_	ZINC132618	290.153
	2	C_14_H_8_BrClFN_3_	ZINC955717	352.594
	3	C_14_H_9_BrFN_3_	ZINC99087	318.149
	4	C_14_H_11_N_3_O	ZINC65031	237.262
	5	C_18_H_12_BrN_3_	ZINC94936	350.219
	6	C_13_H_12_N_6_	ZINC4710712	252.281
	7	C_20_H_17_N_3_OS	ZINC2664933	347.443
	8	C_17_H_18_N_4_O_2_S	ZINC71920558	342.424
	9	C_20_H_17_N_3_	ZINC13863969	299.377
	10	C_22_H_21_FN_6_	ZINC9074069	388.45
	11	C_18_H_20_FN_7_O	ZINC13010674	369.404
	12	C_18_H_21_N_7_O	ZINC22735958	351.414
	13	C_19_H_15_N_3_OS	ZINC117048	333.416
	14	C_14_H_9_ClIN_3_	ZINC955103	381.604
	15	C_15_H_12_BrN_3_	ZINC122234	314.186
	16	C_14_H_9_Cl_2_N_3_	ZINC140100	300.156
	17	C_14_H_10_BrN_3_	ZINC144105	300.159
	18	C_16_H_14_ClN_3_	ZINC48331888	283.762
PI3Kp110β	19	C_27_H_36_F_2_N_4_OS	ZINC20729292	502.675
	20	C_28_H_30_FN_3_O_3_	ZINC36307506	475.564
	21	C_27_H_31_N_3_O_3_S	ZINC9873787	477.63
	22	C_22_H_23_NO_4_	ZINC977288	365.429
	23	C_30_H_42_N_2_O_9_	ZINC68202727	574.671
	24	C_21_H_23_ClFNO_5_	ZINC218287337	423.868
EGFR + PI3K	25	C_24_H_25_ClFN_5_O_3_	ZINC27439698	485.947
	26	C_28_H_21_Cl_2_N_3_O_3_	ZINC20615563	518.4
	27	C_17_H_13_BrN_4_O	ZINC1488208	369.222

EGFR, epidermal growth factor receptor; PI3Kp110β, phosphoinositide 3-kinase p110β isoform.

**Table 2:** Antibodies used for immunocytochemistry analysis.

Target Protein	Primary Antibody	Secondary Antibody
Cyclin D2	Cyclin D2 (1:100)	Alexa Fluor^®^ 555 (1:500)
	Thermo Fisher Scientific,	Thermo Fisher Scientific,
	#AHF0112, Mouse	#A31570, Donkey Anti-Mouse
FOXP1	FOXP1 (1:200)	Alexa Fluor^®^ 555 (1:500)
	Thermo Fisher Scientific,	Thermo Fisher Scientific,
	#PA5-52006, Rabbit	#A21428 Goat Anti-Rabbit
Ki-67	Ki-67 (1:100)	Alexa Fluor^®^ 555 (1:500)
	Thermo Fisher Scientific,	Thermo Fisher Scientific,
	#PA5-19462, Rabbit	#A21428 Goat Anti-Rabbit
MEF2C	MEF2C (1:50)	Alexa Fluor^®^ 555 (1:500)
	Thermo Fisher Scientific,	Thermo Fisher Scientific,
	#PA5-28247, Rabbit	#A21428 Goat Anti-Rabbit

FOXP1, forkhead box P1; MEF2C, myocyte enhancer factor 2C.

## Data Availability

All data generated or analyzed during this study are included in this published article.
